# Effect of Fibroblast Co-culture on In Vitro Maturation and Fertilization of Mouse Preantral Follicles

**Published:** 2011-03-21

**Authors:** Mahmoud Heidari, Abbasali Karimpour Malekshah, Kazem Parivar, Ramezan Khanbabaei, Alireza Rafiei

**Affiliations:** 1 Department of Biology, Science and Research Branch, Islamic Azad University, Tehran, Iran; 2 Department of Anatomy and Embryology, Molecular Cell Biology Research Center, Faculty of Medicine, Mazandaran University of Medical Science, Sari, Iran; 3 Department of Biology, Qaemshahr Branch, Islamic Azad University, Qaemshahr, Iran; 4 Molecular Cell Biology Research Center, Department of Immunology, Faculty of Medicine, Mazandaran University of Medical Science, Sari, Iran

**Keywords:** In Vitro Maturation, Preantral, Follicle, Fibroblast, Co-Culture

## Abstract

**Background:**

The aim of this study was to evaluate fibroblast co-culture on in vitro maturation and fertilization of prepubertal mouse preantral follicles.

**Materials and Methods:**

The ovaries of 12-14 day old mice were dissected and 120-150 μm intact preantral follicles with one or two layers of granulosa cells, and round oocytes were cultured individually in α-minimal essential medium (α-MEM) supplemented with 5% fetal bovine serum (FBS), 100 mIU/ml recombinant follicle stimulating hormone, 1% insulin, transferrin, selenium mix, 100 μg/ml penicillin and 50 μg/ml streptomycin as base medium for 12 days. A total number of 226 follicules were cultured under two conditions: i) base medium as control group (n=113); ii) base medium co-cultured with mouse embryonic fibroblast (MEF) (n=113). Follicular diameters, alone, in addition to other factors were analyzed by student’s t-test and chi-square test, respectively.

**Results:**

The co-culture group showed significant differences (p<0.05) in growth rate (days 4, 6 and 8 of the culture period) and survival rate. However, there was no significant difference in antrum formation, ovulation rate and embryonic development of released oocytes. There were significant differences (p<0.05) in the estradiol and progesterone secretion at all days between the co-culture and control groups.

**Conclusion:**

Fibroblast co-culture increased survival rate and steroid production of preantral follicles by promoting granulosa cell proliferation.

## Introduction

The basic structural and functional unit of ovaries are follicles which provide the necessary microenvironment for oocyte growth and maturation (1). Since preantral follicles are a large potential source of oocytes with potential use for in vitro studies of early folliculogenesis and embryo production, recent research in the field of in vitro maturation (IVM) has focused on the development of preantral follicular culture systems. Therefore, developing a culture system which will result in mature, fertilizable oocytes could be advantageous not only for better understanding of folliculogenesis but also for long-term preservation of female germ cells (2).

To date, different systems have been developed that culture preantral follicles in many species, including mice (3-5), cats (6), cows (7, 8), pigs (9, 10) and humans (11). Additionally, co-culturing with somatic cells is another method used to improve in vitro development of oocytes and embryos (12) since cellular interactions between ovarian germline and somatic cell components are crucial for follicular development and function (13). Certain compounds secreted from somatic cells have been reported to assist with the growth of preantral follicles (14).

Therefore, tissues from different sources such as reproductive and non-reproductive female organs have been used to support the development of different embryos such as human (15), mouse (16), bovine (17) and canine oocytes (18). Use of somatic cells as a feeder layer in co-culture systems has different beneficial effects on embryo development such as the improvement of embryo quality and increasing the rate of embryo development into the blastocyst stage (19). Somatic cells used in the co-culture system have been reported to probably produce unknown promoting factors for embryonic development or delete embryo toxic factors from the culture medium (16). Recent studies have indicated that co-culturing preantral follicles with different somatic cells such as granulosa, cumulus and ovary mesenchymal cells in bovine (14), porcine (20) and murine (12) have a significant influence on the development of preantral follicles. However, the use of a feeder layer to improve IVM of isolated preantral follicles in the co-culture system has received less attention.

Recent studies have used embryonic fibroblast cells for development and maintenance of mouse (21), monkey (22) and human (23) embryonic stem cells. It was also indicated that fibroblast cells as feeder layers may secrete various factors that enhance embryonic development (16). However, since fibroblast cells as a major part of ovarian stromal cells have a close relation to the follicles at different stages and secrete several cytokines such as leukemia inhibitory factor, steel factor and basic fibroblast growth factor (bFGF) (18), we hypothesized that co-culturing with mouse embryonic fibroblast (MEF) cells may promote preantral follicle development. The effect of MEF co-culture on in vitro maturation of preantral follicles has not been studied previously in any species. Thus, to improve culture conditions and to develop appropriate culture systems with the idea of stimulating preantral follicle growth, the present study was conducted to investigate the effect of MEF on in vitro growth of mouse preantral follicles and embryonic development of their oocytes.

## Materials and Methods

This project was approved by the Islamic Azad University Science and Research Branch.

### Chemicals

All reagents were obtained from Sigma-Aldrich (Germany) unless otherwise specified.

### Animals

Male and female NMRI mice were housed and bred in the central animal house of the Mazandaran University of Medical Science under a 12 hour light/12 hour dark regime at 22-24°C, with adequate food ad libitum.

### Preantral follicle isolation

Female mice, 12-14 days old, were killed by cervical dislocation and their ovaries dissected free of fat and mesentery. Ovaries were immediately transferred to dissection medium that consisted of α-minimal essential medium (α-MEM, Gibco, UK) supplemented with 10% fetal bovine serum (FBS, Gibco, UK), 100 μg/ml penicillin and 50 μg/ml streptomycin under mineral oil to prevent evaporation and severe pH and temperature fluctuation. Preantral follicles from ovaries were isolated by mechanical dissection under a stereomicroscope, using 27-gauge needles to ensure that the follicular structure remained intact. Isolated follicles were selected according to the following criteria: 1. intact follicle with one or two layers of granulosa cells and some adhering theca cells; 2. visible, round and central oocyte; and 3. follicle diameter between 120-150 μm. However, if the follicle had a non-spherical structure, two oocytes or diameter above or below the range of 120-150 μm, they were not selected for culture. All selected follicles were pooled and randomly divided between the study culture conditions. Then, isolated follicles were transferred to fresh culture medium.

### Preantral follicle culture

Isolated preantral follicles were cultured individually in 60 mm petri dishes (Falcon, Becton Dickinson, Belgium) that contained 19×30 μl droplets of α-MEM (Gibco, UK) supplemented with 5% FBS, 100 mIU/ml recombinant follicle stimulating hormone (rFSH or Gonal-f, Serono, Switzerland), 1% insulin, transferrin and selenium mix (ITS mix: 5 μg/ml, 5μg/ml and 5 ng/ml, respectively; Gibco, UK), 100 μg/ml penicillin and 50 μg/ml streptomycin as base medium under mineral oil and incubated at 37°C in a humidified atmosphere of 5% CO2 in air for 12 days (5).

In the dishes, half of the medium was sampled from each droplet every two days without damaging the follicle. It was replaced by 15 μl of fresh pre-equlibrated medium. All 15 μl droplets, except those from non-proliferating follicles in one culture dish were pooled and stored at -20°C until analysis.

### Measurement of follicular diameter

Measurement of follicle diameter was assessed with a precalibrated ocular micrometer at ×100 magnification every 48 hours during the culture period.

From day four, we could not measure the exact diameter of the growing follicles because of the irregular follicular shape that resulted from granulosa cells piercing the basement membrane and the onset of creating a monolayer around the follicle; thus we measured approximate follicle diameters. The survival rate of the follicles was verified by evaluation of follicle morphology under an inverted microscope. Follicle survival in culture was considered positive as long as an oocyte remained surrounded by granulosa cells attached to the culture dish.

### In vitro ovulation induction

**Fig 1:**
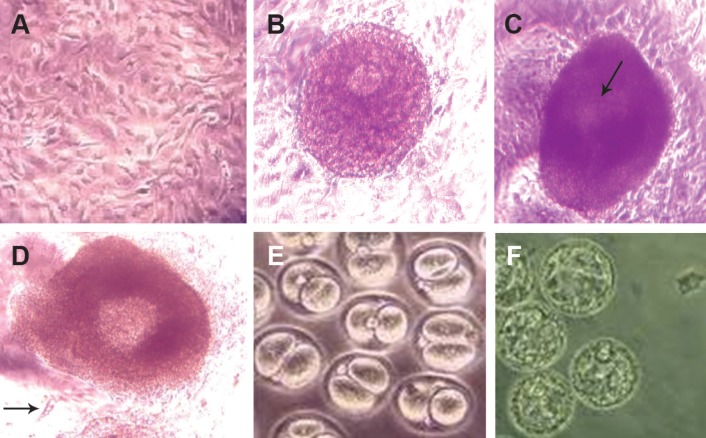
In vitro maturation of preantral follicle from 14 day old mice. A. Fibroblast monolayer for co-culturing preantral follicles (scale bar: 50 μm). B. Preantral follicle after 4 days of co-culture with fibroblast cells. Germinal vesicle stage oocyte surrounded by several layers of granulosa cells (scale bar: 50 μm). C. Follicle co-cultured with fibroblast cells for 12 days and developed antrum cavity (arrow) (scale bar: 150 μm). D. Released cumulus oocyte complex 16-24 hours post hCG (arrow) (scale bar: 100 μm). E. Embryos at 2-cell stage (scale bar: 50 μm). F. Embryos at blastocyst stage (scale bar: 50 μm).

On day 12 of the culture, ovulation was induced by collecting the total volume of droplets and the addition of fresh medium supplemented with 1.5 IU/ ml human chorionic gonadotropin (hCG; Organon) to the droplets. Mucification of the cumulus oocyte complexes (COC) was observed 14-16 hours later under inverted microscope (Fig 1D). Since the oocytes were not denuded, the exact oocyte nuclear maturity (MII) potential could not be scored.

### In vitro fertilization and culture of oocytes

A sperm suspension was prepared using spermatozoa collected from the cauda epididymis of mature F1 males and preincubated at a concentration of 10-15 × 106 cells/ml for 90 minutes in 500 μl of HTF medium that contained NaCl (5.935 g/1), NaHCO3 (2.1 g/1), α-Dglucose (0.5 g/1), KC1 (0.35 g/1), KH2PO4 (0.05 g/1), sodium pyruvate (0.036 g/1), penicillin- G (0.06 g/1), streptomycin sulphate (0.05 g/1), CaCl2-2H2O (0.3 g/1), EDTA (0.021 g/l MgSO47H2O (0.024 g/1), sodium lactate syrup (3.2 ml of 60% syrup) and phenol red (0.01 g/1) supplemented with 4 mg/ml bovine serum albumin (BSA) fraction v (Sigma) to induce sperm capacitation. The in vitro released mucified COCs were placed in 100 μl (10 COCs/drop) fertilization droplets of HTF medium with 4 mg/ml BSA and 106/ml spermatozoa. After 4-5 hours, oocytes were washed and cultured in 20 μl droplets (10 oocytes/drop) of T6 medium with 4 mg/ml BSA under mineral oil at 37ºC in an atomosphere of 5% CO2 in air for five days until the blastocyst stage. The developmental stages of inseminated oocytes were determined by morphological evaluation every 24 hours under an inverted microscope. Fertilization rate was scored as the percentage of 2-cell embryos observed 24 hours after insemination (Fig 1E-F).

### Mouse embryonic fibroblast feeder layer

We prepared MEF according to Hatoya et al. with some modification (18). Briefly, fetuses were collected from female NMRI mice at days 12 to 13 of pregnancy and washed thoroughly in Dulbecco’s phosphate-buffer saline (PBS). The head and liver were removed, samples cut into small pieces and cultured in α-MEM supplemented with 10% FBS, 100 IU/mL penicillin and 100 mg/ml streptomycin in a humidified atmosphere of 5% CO2 in air at 37ºC. Primary fibroblasts were cultured until confluent and proliferated through two subsequent passages. For preparation of a feeder layer, MEF was inactivated by 10 μg/ml mitomycin C (Kyowa, Tokyo, Japan) for 3 hours in a humidified atmosphere of 5% CO2 in air at 37ºC, and washed five times in PBS. These cells were subsequently frozen at -80ºC in a volume of 106 cells/ml in the freezing medium that contained α-MEM supplemented with 100 IU/ml penicillin and 100 mg/ml streptomycin, 60% FBS and 10% DMSO (dimethyl sulfoxide). Frozen cells were thawed in water at 37ºC, cultured and plated at a density of 1×105 cells/ml one day before use. On the day of culture, the fibroblasts had made a feeder layer in the culture droplets (Fig 1A).

### Experimental design

To evaluate the supporting effect of fibroblast coculture, preantral follicles were cultured in two culture conditions. Follicle diameter, survival rate, antrum formation and embryonic development were studied in a total number of 226 intact preantral follicles with diameters between 120-150 μm. Two follicle culture conditions were studied and the experiments were repeated three times per group: i. follicle culture in base medium as control group (n=113) and ii. follicle culture in the base medium co-cultured with fibroblasts (n=113).

### Assessment of steroid hormone

In the ~15 μl medium droplets, which were sampled and subsequently replaced by fresh medium, the following secretory products were measured: estradiol and progesterone. By measuring estradiol and progesterone, we wished to obtain information on the steroidogenic pathways functioning throughout the culture period and on the differentiation of the granulosa cells in culture. Every other day, all ~15 μl samples from surviving follicles of each group were pooled.

Estradiol and progestrone were measured using commercially available radio-immunoassay kits that included the IBL (Germany) kit with a sensitivity of 9.7 pg/ml and a total precision of <10% (% coefficient of variation; CV) and the Demeditec (Germany) kit with a sensitivity of 0.04 ng/ ml and a total precision of <10 % CV, respectively.

### Statistical analysis

Data are presented as mean ± standard deviation (SD). Statistical analysis was performed using the Statistical Package for Social Scieneces (SPSS version 15). Follicular diameters were analyzed by student’s t-test. Survival rate, antrum formation, COC recovery or antrum formation and embryonic development of fertilized oocytes in the two groups were compared by the chi-square test. P<0.05 was considered statistically significant.

## Results

### Evaluation of follicles in culture

On day two of culture, spindle-shaped cells originating from the surface of the follicle attached themselves to the dish and proliferated, with the formation of a monolayer that surrounded and attached the follicle to the dish. By day four, follicles attached to the dish. Granulosa cells proliferated and broke through the basal membrane, spreading over the basal membrane and the monolayer already formed the initial follicular monolayer that surrounded and attached the follicle to the dish (Fig 1B). Most follicles lost their follicular structure and developed a 'diffuse appearance' on day four of culture. The follicles reached a 'diffuse' pattern by day 10. Antrumlike cavity formation (indicated by the appearance of lucid patches within the granulosa cell mass) was recognized from day eight onwards. By day 12, clearly visible and well developed antral-like cavities were recognized in the fully grown antral follicles (Fig 1C). Follicles showing a spontaneous oocyte release were maintained for further evaluation.

### Follicular growth rates

Follicle diameters were estimated every two days to evaluate the effects of fibroblast co-culture on the development of preantral follicles during the 12 day culture period. The respective diameters of the follicles (mean ± SD) in the control and co-culture groups were as follows: 138 ± 7.7 μm and 137 ± 10.1 μm (day 0); 173.3 ± 12.3 μm and 191.3 ± 15.9 μm (day 2); 233.3 ± 38.6 μm and 276 ± 52.4 μm (day 4); 323.3 ± 40.3 μm and 386.6 ± 58.1 μm (day 6); 394 ± 64.8 μm and 455.3±71.7 μm (day 8); 460 ± 89 μm and 483.3 ± 64.5 μm (day 10); 493.3 ± 41.7 μm and 526.6±69.3 μm (day 12), respectively.

**Fig 2:**
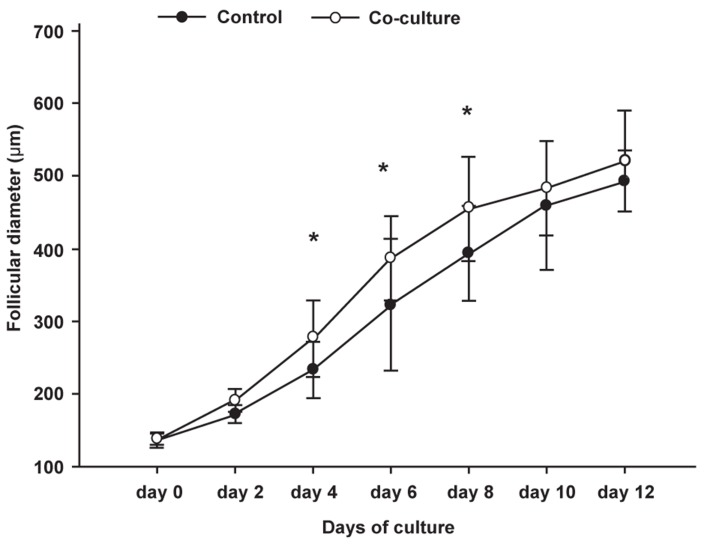
Comparison of the growth rates of follicles (mean follicular diameter ± SD) in the control and co-culture groups. Follicular diameters in the co-culture group were significantly different (*p<0.05) at days 4, 6 and 8 of culture when compared to the control group.

A comparison of the follicular growth rates in the control and co-cultured groups is shown in figure 2 (significant difference between groups at p<0.05). As shown in figure 2, follicular diameter increased after 12 days of culture in both groups; but coculturing had a positive, significant effect on follicular diameter on days 4, 6 and 8 of the culture period. Therefore, fibroblast co-culture stimulated the growth of preantral follicles by granulosa cell proliferation at the middle of the culture period, the time when follicles lose their initial structure and proceed to the next step as a diffuse stage.

### Survival rates

A normal follicular structure contains a centrally located oocyte surrounded by several layers of granulosa cells within the basal layer. In the present study, follicles were considered viable if they retained a normal structure with close contact between the oocytes and the surrounding granulosa cells. Follicles that failed to survive were defined as those that had lost their oocytes, failed to increase in diameter and became necrotic in appearance. Follicular survival rates in the two groups after 12 days of culture were between 80.5%-88.5% as shown in Table 1. Follicles in the co-culture group had a higher survival rate (88.5 ± 1.4) compared to the control group (80.5 ± 2.3) at p<0.05 (Table 1).

### Antrum formation rates

Antrum-like cavities were recognized from day eight onwards. Antral rates of both groups were between 85.7%-87%. Despite significant differences between the two groups in survival rates, however, there was no significant difference in antrum formation rates between the co-culture and control groups as shown in table 1.

### Ovulation rates

At the end of the culture period, hCG (1.5 IU/ml) was supplemented to induce ovulation. After 18- 24 hours, cumulus oocyte complexes (COC) were counted for evaluation of ovulation rate or COCs recovery (Table 1). Ovulation rates in the two groups were between 80.2%-84 %. There was no significant difference in ovulation rates between the two groups as shown in table 1.

### In vitro fertilization and embryonic development of in vitro matured oocytes

In vitro fertilization evaluated the fertilizing potential as well as embryonic development of in vitro matured oocytes. Since the oocytes obtained from COCs were not denuded, the exact fertilization potential could not be scored.

**Table 1:** Effect of fibroblast co-culture on in vitro maturation and embryonic development of oocytes derived from preantral follicles


	No. (%) of follicles			Percent (%) of oocytes developed to		
Groups	Cultured follicles	Survival rates	Antral rates	Ovulation rates	2-cell	Morula	Blastocyst
**Control**	113	91 (80.5)	78 (85.7)	73 (80.2)	30 (41.1)	15 (21.7)	9 (12.3)
**Co-culture**	113	100 (88.5)*	87 (87)	84 (84)	38 (45.2)	19 (22.6)	10 (11.9)


*Significant differences are compared with control group (p<0.05).

**Table 2:** Production of estradiol (pg/ml) and progesterone (ng/ml) in pooled media during culture period of preantral follicles. There are significant differences (p<0.05) in both estradiol and progesterone secretion at days 4, 6, 8, 10, 12 and days 10 and 12, respectively between the co-culture and control groups


Estradiol (pg/ml)						Progesterone (ng/ml)			

**Group**	Day 4	Day 6	Day 8	Day 10	Day 12	Day 6	Day 8	Day 10	Day 12
**Control**	31.6	226	868	1642	3449	-	-	12.7	16.7
**Co-culture**	47*	243*	934*	1882*	3708*	7.2	13.4	15 *	22*


*Significant differences are compared with control group (p<0.05).

Therefore, the percentage of 2-cell embryos observed 24 hours after fertilization was assumed to be the fertilization rate. As shown in Table 1, the fertilization rates of both groups were 41.1%-45.2 % and the percentages of morula and blastocyst embryos were between 21.7%-22.6% and 11.9%- 12.3%, respectively. There was no significant difference (p<0.05) between control and co-culture groups regarding embryonic development.

### Assessment of steroid hormones

Production of estradiol (pg/ml) and progesterone (ng/ml) are shown in table 2. The production of estradiol increased progressively up to day 12. On this day, the production of estradiol reached 3708 pg/ml in the co-culture group. There were significant differences (p<0.05) in estradiol secretion on days 6, 8, 10 and 12 between the co-culture and control groups. Progesterone production remained below the sensitivity of the radioimmunoassay up to days 6 and 10 for the co-culture and control groups, respectively. Basal progesterone production increased moderately after this day to a level of 22 ng/ml in the co-cultured group. Progesterone concentrations were also significant (p<0.05) in the co-culture group on days 10 and 12 compared to the control group.

## Discussion

We established follicle cultures to study essential factors for oocyte development during in vitro folliculogenesis (24) in a small microdroplet. This is advantageous in that it permits regular observation of follicle and oocyte growth in addition to providing access to the secretory products of single follicles without disturbing current growth (25).

In the present study, early preantral follicles were cultured for 12 days in co-culture conditions to evaluate whether MEF co-culture could enhance follicular growth rates as well as in vitro fertilization and embryonic development of in vitro matured oocytes.

The results of this study showed that the co-culture group had a significant (p<0.05) growth rate (on days 4, 6 and 8 of the culture period) and survival rate when compared to the control group. From days 4 until 8, granulosa cells proliferated and their protrusion through the basement membrane led to the formation of large preantral follicles (26). It has been determined that FSH stimulates proliferation and differentiation of the granulosa cells in addition to inducing FSH and luteinizing hormone (LH) receptors in them (27). On the other hand, the function of gonadotropins in follicular development are indirectly regulated by expression of hepatocyte growth factor (HGF), kit ligand (KL) and fibroblast growth factors (FGF) which are secreted from fibroblast cells (28). Thus, MEF cells may contribute to the growth and survivability of preantral follicles by providing growth factors such as bFGF, steel factor and leukemia inhibitory factor (18). These results support the findings of previous studies, wherein they obtained significantly higher growth and survival rates in preantral follicles co-cultured with different somatic cells such as cumulus, granulosa, ovary mesenchymal and oviductal epithelial cells in different species (12, 14, 20). Ramesh et al. have shown that buffalo preantral follicles co-cultured with cumulus, granulosa and ovary mesenchymal cells had better development and survivability in vitro compared to a control group. They suggested that ovarian mesenchymal cells (like fibroblast cells) may contribute to the growth and survivability of preantral follicles by providing factors such as extracellular matrix proteins, basement membrane components, high molecular mass proteins, transforming growth factor (TGF), keratinocyte growth factor (KGF) and HGF. They also suggested that granulosa cells might enhance growth and survivability of preantral follicles by producing activin, inhibin, thecal differentiation factor and fibronectin (14).

The results of this study also showed no significant difference between the two groups in antrum formation and ovulation rates of follicles that survived. However, compared to the findings of a study (12) that co-cultured mouse preantral follicles with cumulus cells which reported a survival rate of 72% and 35.5% antral rate in the co-culture group, our results were 88.5% and 87%, respectively, in the fibroblast co-culture group. These findings suggest that co-culture of preantral follicles with fibroblast cells may be more beneficial for their development.

Our results showed no significant difference in embryonic development between the co-culture and control groups. This result suggests that the co-culture system used in this study has no remarkable effect on embryonic development of oocytes from in vitro matured preantral follicles, however it might be caused by insufficient cytoplasmic maturation. A similar result was reported by Haidari et al. who found no difference in the rates of fertilization and subsequent development to the blastocyst stage between the oocytes derived from cumulus co-cultured preantral follicles and those derived from control preantral follicles (12). However some studies demonstrated that the co-culture system enhanced embryonic development of in vitro matured oocytes in different species. For example, Hatoya et al. showed that co-culture with embryonic fibroblast cells enhanced nuclear and cytoplasmic maturation of in vitro matured canine oocytes (18). Nasr-Esfahani et al. also reported that co-culture of bovine oocytes with vero cells during in vitro maturation enhanced the potential for cleavage and production of higher quality blastocysts (17).

Since steroid hormones might be important regulators of crucial changes in oocyte cytoplasm for normal fertilization, measurements of estradiol and progesterone in conditioned media from cultured follicles can provide precise information on the functionality of the culture condition (25). As shown in table 2, the production of estradiol increased in a linear fashion up to day 12 in both groups. There were significant differences in estradiol secretion on days 4 up to 12 between the co-culture and control groups. However progesterone production remained below the sensitivity of the radioimmunoassay technique up to days 6 and 10 in the co-culture and control groups, respectively. Cortvrindt et al. also reported progesterone detection from day 8 onward, which indicated a high progesterone concentration on days 14 and 16 of the culture period (29). This significant production of steroid hormones could be the outcome of supportive effects of the fibroblast co-culture system on in vitro growth and maturation of preantral follicles.

## Conclusion

Our results suggest that fibroblast co-culture increases the growth and survival rate of cultured preantral follicles in a significant manner by enhancement of granulosa cell proliferation; however, the co-culture had no effect on embryonic development of the ovulated oocyte. This research investigated, for the first time, the effect of MEF co-culture on preantral follicles. However, more research is necessary for the improvement of in vitro maturation of preantral follicles.
